# Diagnostic Pitfalls in Peritoneal Carcinomatosis: A Case of Pseudomyxoma Peritonei

**DOI:** 10.7759/cureus.85474

**Published:** 2025-06-06

**Authors:** Maksim Isachanka, Ilya Tarasau, Olga Matylevich, Siarhei Mavrichev, Alena Dalamanava, Dmitry Rovski, Aliaksei Kurchankou, Mariya Galka, Mikalay Kurchankou

**Affiliations:** 1 Internal Medicine, St. Elizabeth's Medical Center, Boston, USA; 2 Gynecologic Oncology, N. N. Alexandrov National Cancer Centre of Belarus, Minsk, BLR; 3 Laboratory of thoracic oncopathology, N. N. Alexandrov National Cancer Centre of Belarus, Minsk, BLR; 4 Molecular Genetic and Pathology, Republic Reference Centre for Molecular Genetic and Pathological Diagnostics of Lymphoproliferative Diseases, Malignant Soft Tissue, Bone and Central Nervous System Tumors, Minsk, BLR; 5 Radiology, N. N. Alexandrov National Cancer Centre of Belarus, Minsk, BLR

**Keywords:** cytoreductive surgery, gynecologic oncology, hyperthermic intraperitoneal chemotherapy, mucinous neoplasms, pseudomyxoma peritonei

## Abstract

Pseudomyxoma peritonei (PMP) is a rare pathological condition posing significant diagnostic and management challenges. This article presents a clinical case of a 58-year-old female who was initially diagnosed with stage III primary peritoneal carcinoma. Following neoadjuvant chemotherapy, a diagnostic re-evaluation was performed with histopathological and immunohistochemical review of biopsy specimens, which led to a revised diagnosis of mucinous carcinoma with features consistent with PMP. The patient subsequently underwent complete cytoreductive surgery (CRS) followed by hyperthermic intraperitoneal chemotherapy (HIPEC). She has remained disease-free for 12 months post-operatively.

This case illustrates the crucial role of pathological assessment in guiding treatment for PMP and demonstrates favorable long-term outcomes with aggressive CRS and HIPEC in appropriately selected patients.

## Introduction

Pseudomyxoma peritonei (PMP) is a rare clinical syndrome characterized by the accumulation of mucinous ascites within the peritoneal cavity and the peritoneal implantation of mucin-producing tumor cells. This pathology was first described by Carl F. Rokitansky in 1842 [1]. Later, in 1884, Werth classified PMP as mucinous tumors associated with the ovaries [2], whereas Frankel, in 1901, proposed an alternative origin from a ruptured appendiceal mucocele [3].

Current research confirms that in the vast majority of cases, the source of PMP is a low-grade appendiceal mucinous neoplasm (LAMN) [4]. These tumors produce abundant extracellular mucin, predominantly composed of high-molecular-weight glycoproteins such as MUC2 [5]. The mucinous peritoneal deposits in PMP can be either acellular or contain neoplastic epithelial cells with low- or high-grade histologic features, a distinction that holds significant prognostic importance. The accumulation of mucinous ascites within the peritoneal cavity in PMP can provoke a secondary peritoneal reaction, including fibrosis and the formation of adhesions.

PMP occurs more frequently in women, particularly over the age of 40, and the most common presenting symptom is a progressive increase in abdominal girth [6]. Among imaging modalities, computed tomography (CT) holds the greatest diagnostic value and is recognized as the "gold standard" for detecting PMP [7]. However, paracentesis or needle aspiration are generally ineffective for diagnosis and management due to the high viscosity and density of the mucinous material [8].

Currently, the cornerstone of PMP therapy is cytoreductive surgery (CRS) aimed at removing the primary tumor site and achieving maximal debulking of the mucinous content [9]. Despite the disease's propensity for recurrence, the implementation of a comprehensive approach including CRS and hyperthermic intraperitoneal chemotherapy (HIPEC) has led to significant improvements in long-term outcomes. Long-term remissions, lasting 10 years or more, are reported in some patients [10].

Five-year survival rates for patients with PMP vary widely, ranging from 6.7% for peritoneal mucinous carcinomatosis (PMCA) to 84% for disseminated peritoneal adenomucinosis (DPAM), depending on the morphological subtype, its grade of differentiation, and the completeness of surgical cytoreduction [11]. Thus, PMP represents a complex clinical and oncological challenge requiring a multidisciplinary approach, early diagnosis, and personalized selection of treatment strategies.

## Case presentation

Clinical findings and investigations

A 58-year-old female patient was admitted to the N.N. Alexandrov National Cancer Centre of Belarus. Her general performance status was assessed as good (Eastern Cooperative Oncology Group, ECOG performance status 0). She was initially diagnosed with primary peritoneal carcinoma, cT3cN0M0 stage III (FIGO), based on the findings of a diagnostic laparoscopy and biopsy performed on September 29, 2023, at her local oncology hospital. The Peritoneal Cancer Index (PCI) score was 14.

The patient underwent neoadjuvant systemic chemotherapy comprising six cycles of paclitaxel (300 mg) and carboplatin (700 mg), the last of which was completed on February 8, 2024. Upon admission to our institution, a comprehensive review of the original histologic slides was conducted to reassess the diagnosis and guide further management. Based on the results of the histological review, a diagnosis of mucinous carcinoma with a characteristic pattern of tumor spread consistent with PMP was established.

**Figure 1:**
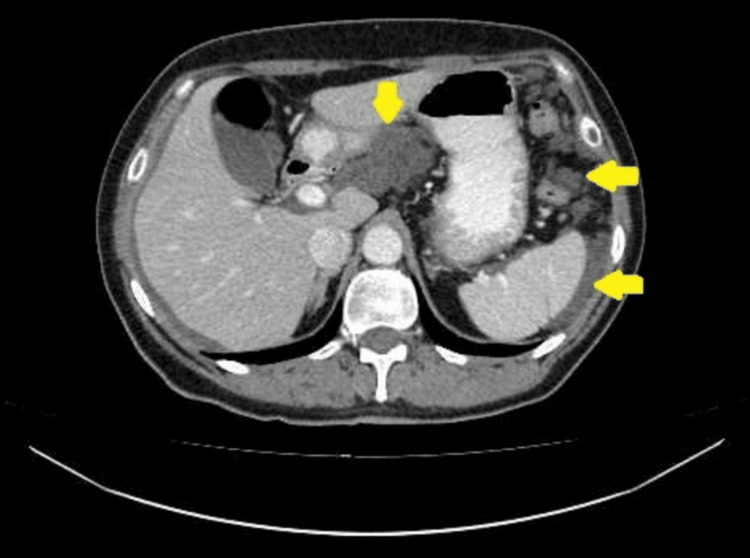
CT imaging of peritoneal metastases and local fluid accumulation in the abdomen and pelvis. Arrows (from top to bottom accordingly) indicate metastases in the small omentum, metastases in the left lateral canal, and fluid accumulation in the perisplenic space.

**Figure 2:**
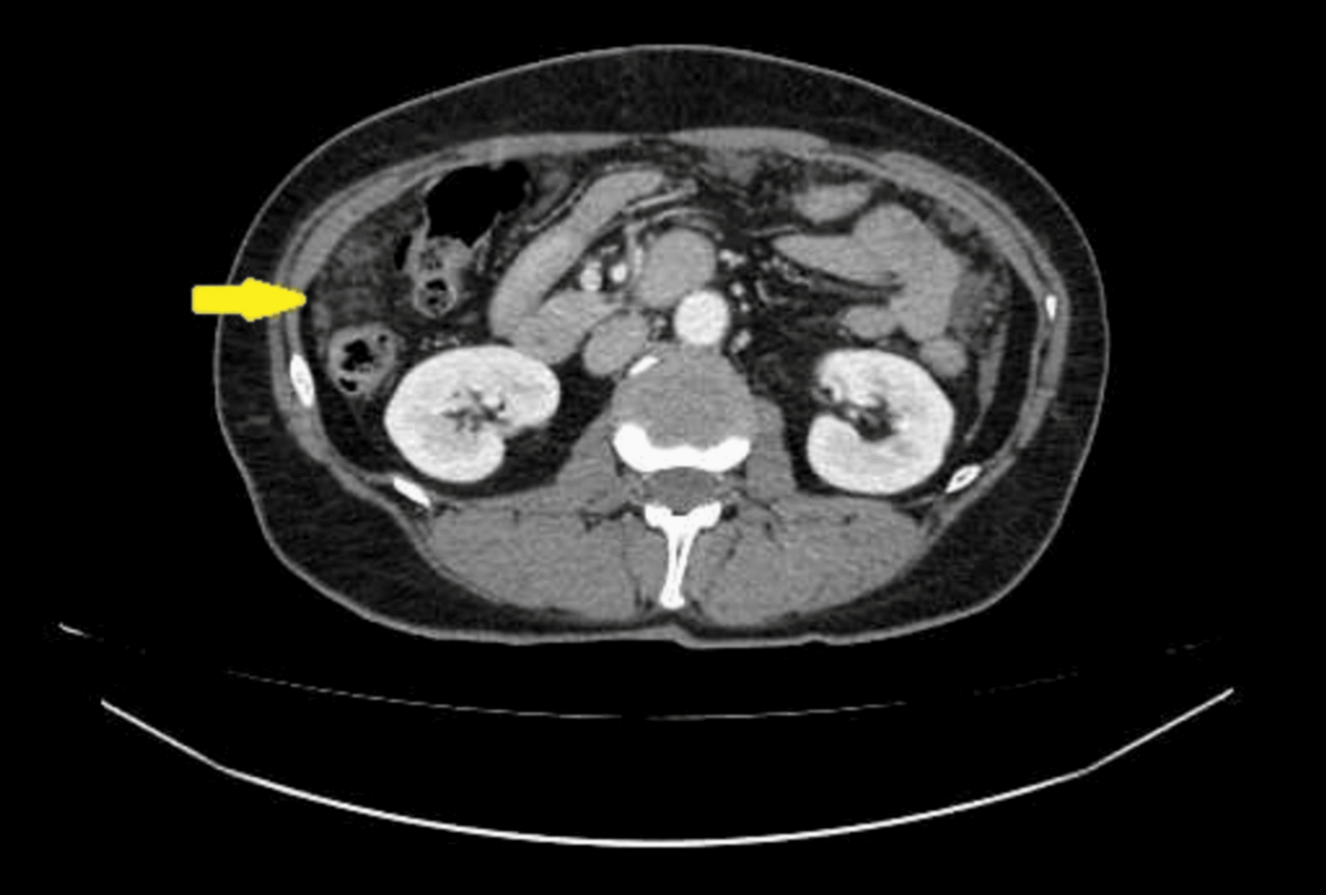
CT imaging of peritoneal metastases and local fluid accumulation in the abdomen and pelvis. The arrow shows metastases in the right lateral canal.

**Figure 3:**
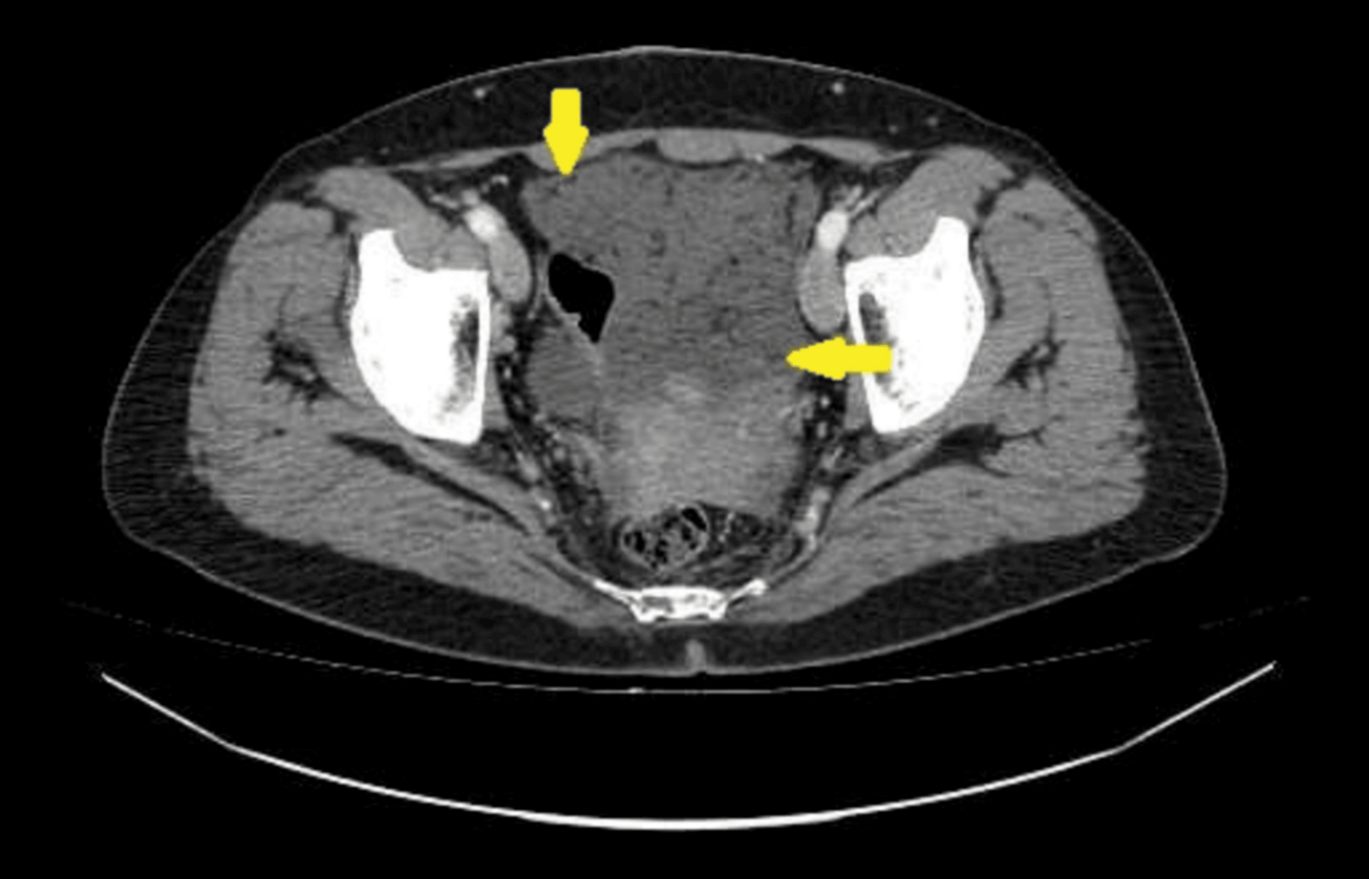
CT imaging of peritoneal metastases and local fluid accumulation in the abdomen and pelvis. Arrows show metastases in the greater omentum.

**Figure 4:**
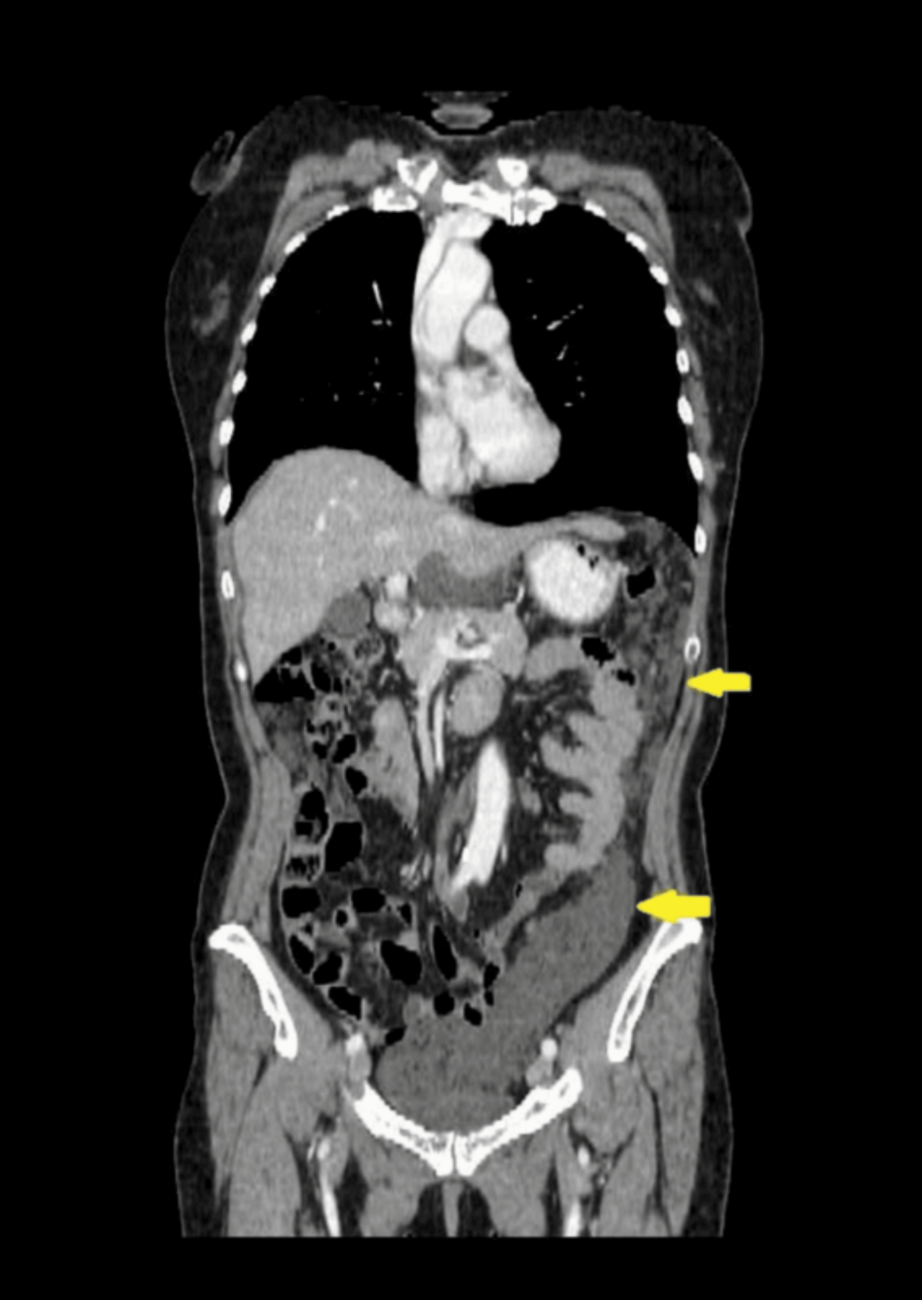
CT imaging of peritoneal metastases and local fluid accumulation in the abdomen and pelvis. Multiplanar reconstruction in the coronal plane. Arrows show metastases in the greater omentum.

CT of the chest, abdomen, and pelvis with intravenous contrast administration revealed multiple collections of low-density fluid (suggestive of mucin) in the perihepatic and perisplenic spaces, as well as within the pelvic cavity. Conglomerate peritoneal deposits were detected in the greater and lesser omentum and the lateral gutters, indicating extensive peritoneal involvement. No other pathological changes were identified in the organs of the chest or abdomen. Lymph nodes within the scanned area were not enlarged. No signs of bone destruction or osteoblastic activity were detected (Figures 1-4).

Serum biochemical analysis showed normal levels of tumor markers: CA 125 - 14.27 U/mL, CA 19-9 - 14.09 U/mL, and carcinoembryonic antigen (CEA) - 2.01 ng/mL. Based on the combination of clinical, laboratory, and morphological data, a provisional diagnosis was established: mucinous ovarian neoplasm with PMP-type spread, stage cT3cN0M0 (FIGO IIIC). Considering the histological features of the tumor, its extent of spread, and the prior chemotherapy, the multidisciplinary tumor board decided to proceed with CRS, aiming for complete cytoreduction (Completeness of cytoreduction score, CC0), followed by hyperthermic intraperitoneal chemotherapy (HIPEC) as the definitive treatment phase.

Surgical intervention and hyperthermic intraperitoneal chemotherapy (HIPEC)

**Figure 5:**
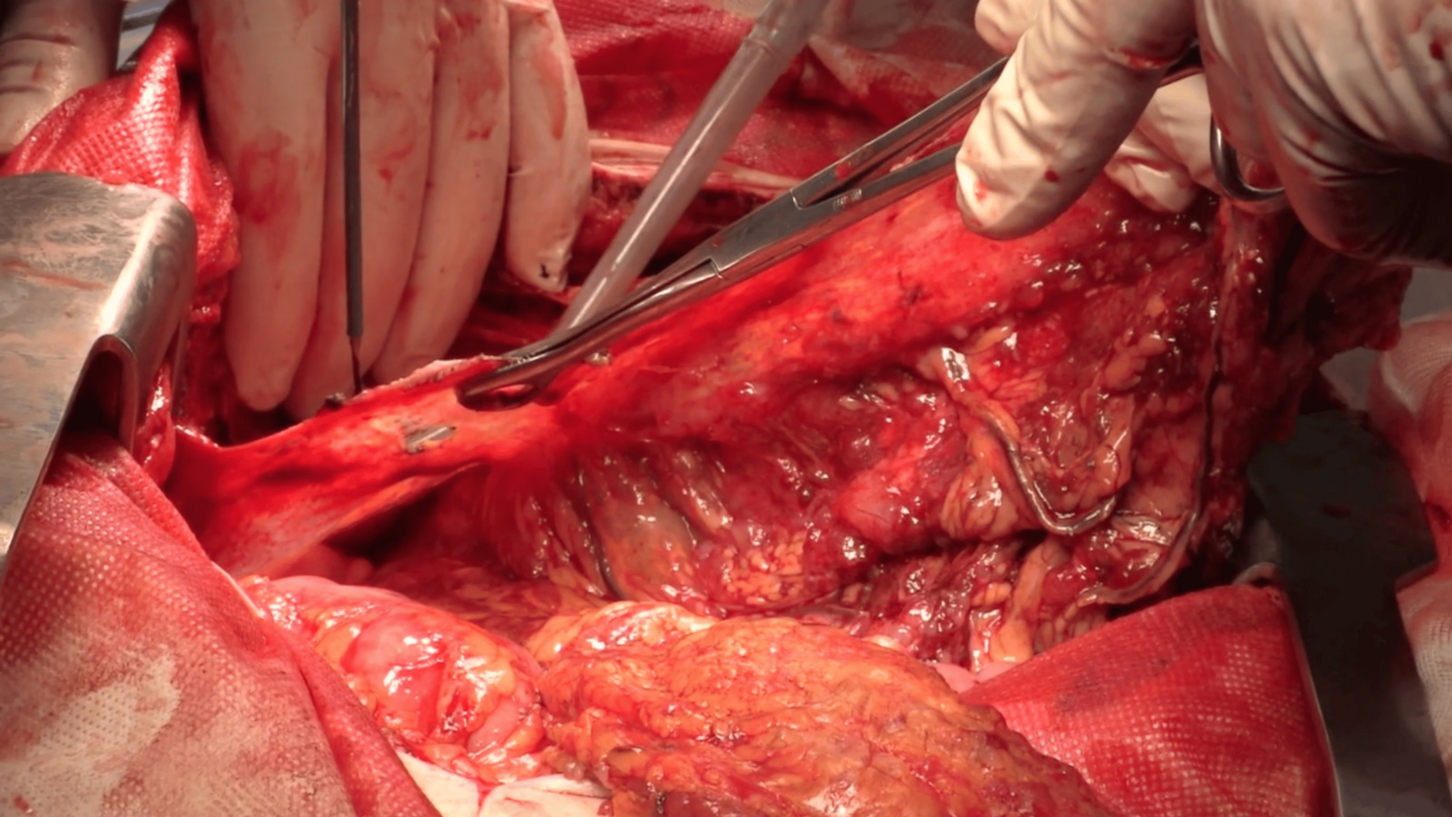
Tumor conglomerate in the pelvis.

The patient underwent a full midline laparotomy extending from the pubic symphysis to the xiphoid process. During intraoperative exploration of the abdominal cavity, multiple tumor deposits were identified: on the surface of the right hemidiaphragm, the liver capsule (Glisson's capsule), and in the region of the gallbladder with involvement of the round ligament of the liver. Massive tumor involvement of the greater omentum was also found; it had descended into the pelvic cavity and was adhered to the uterus and adnexa, forming a single conglomerate mass (Figure 5). Additionally, multiple confluent peritoneal deposits were visualized in the pelvic and abdominal regions, ranging in size from 10 to 150 mm. Based on the identified extent of tumor spread, a CRS was performed, including: total hysterectomy with bilateral salpingo-oophorectomy, pelvic peritonectomy, omentectomy with en bloc resection of a segment of the ileum, right paracolic gutter peritonectomy, cholecystectomy, appendectomy, resection of the lesser omentum, lymph node dissection along the hepatoduodenal ligament, stripping of the right hemidiaphragm peritoneum, and removal of tumor deposits from the small and large bowel mesentery. Thus, complete cytoreduction was achieved with the removal of all visible tumor deposits, corresponding to a completeness of cytoreduction (CC) score of 0 (CC0).

For the HIPEC procedure, three drains equipped with temperature probes were placed into the abdominal cavity to allow continuous monitoring of temperature and solution circulation. After watertight closure of the abdominal wall, the HIPEC procedure was performed using the ThermoChem HT-1000 system. The intraperitoneal temperature was maintained at 41-42 °C for 60 minutes. Cisplatin was used as the chemotherapeutic agent at a dose of 170 mg (corresponding to 100 mg/m^2^ of the patient’s body surface area). The total operative time was 9 hours and 15 minutes. Estimated intraoperative blood loss was approximately 1000 mL.

Postoperative course

**Figure 6:**
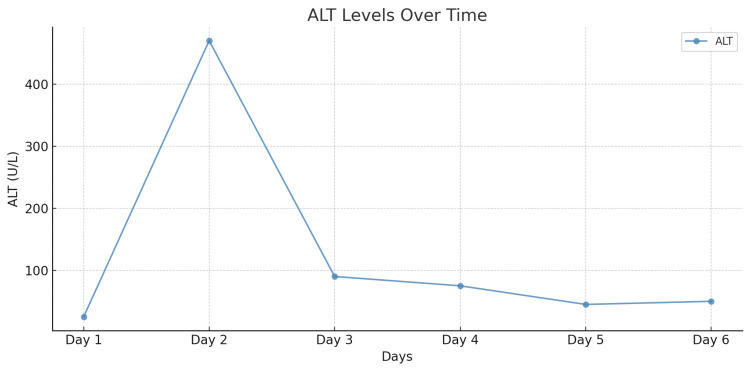
ALT level dynamics over a 14-day period. ALT: Alanine transaminase

**Figure 7:**
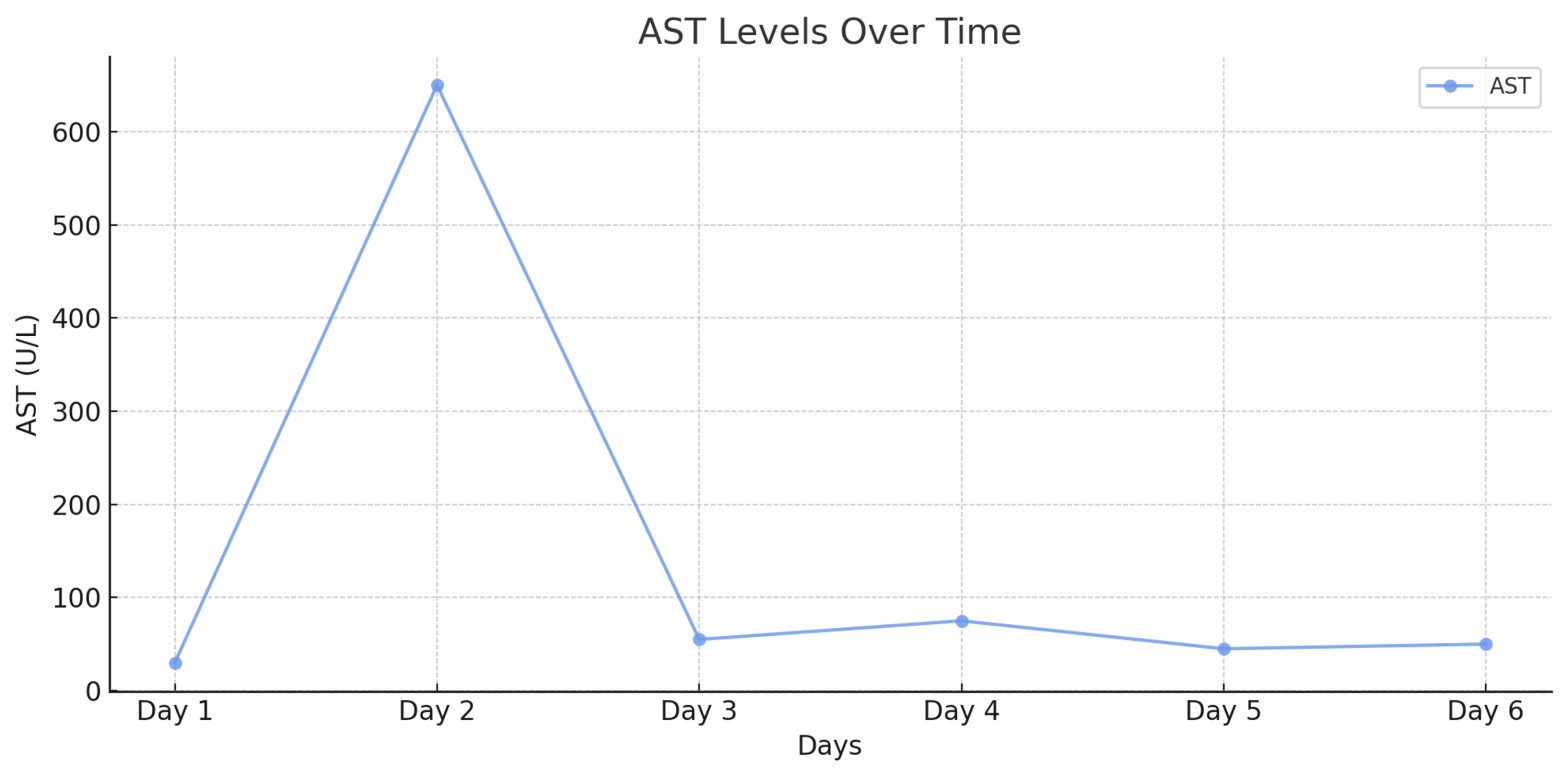
AST level dynamics over a 14-day period. AST: Aspartate aminotransferase

**Figure 8:**
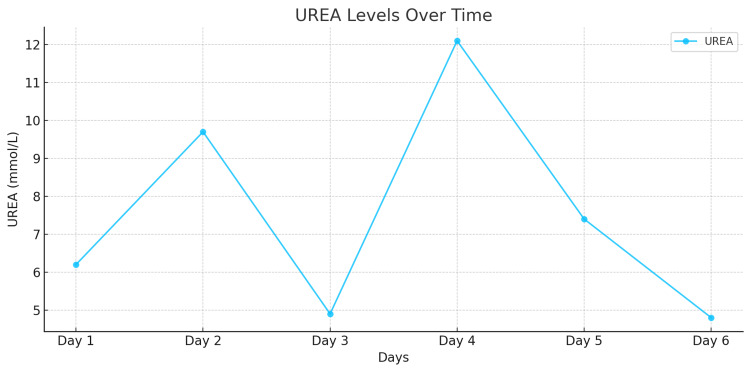
Dynamics of serum urea levels in the patient during a 14-day period.

**Figure 9:**
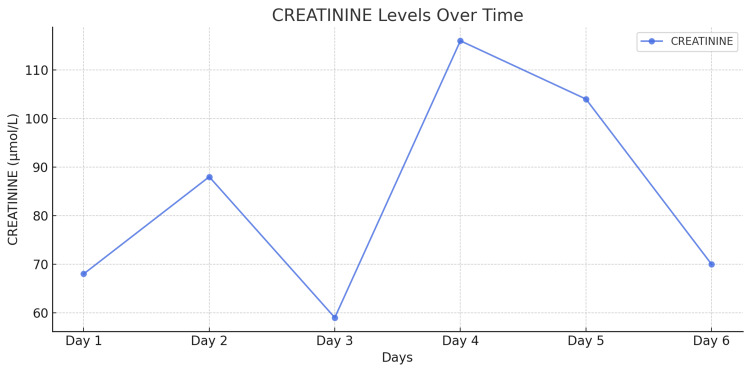
Dynamics of serum creatinine levels in the patient during a 14-day period.

In the early postoperative period, a transient elevation was noted in the levels of liver enzymes-alanine aminotransferase (ALT) and aspartate aminotransferase (AST)-which reached peak values on the first postoperative day (Figures 6-7). However, with supportive management, their levels gradually normalized, indicating recovery of liver function. Urea and creatinine levels reached their maximum values on postoperative day five but remained within the reference range, ruling out significant renal dysfunction (Figures 8-9). Subsequently, these levels stabilized and normalized. The patient was discharged on postoperative day 14 in satisfactory condition, with no signs of early postoperative complications.

Pathological findings

In the pathology laboratory, a comprehensive gross and microscopic evaluation was performed on the surgical specimen, which included the uterine adnexa, the vermiform appendix, and marked fragments from various anatomical sites: peritoneum of the right paracolic gutter, diaphragmatic peritoneum, Morison’s pouch, the round ligament and capsule of the liver, the lesser and greater omentum, segments of the small intestinal wall, and lymph nodes from the hepatic hilum.

Grossly, both ovaries measured 3.0 cm and 3.5 cm in their greatest dimension and were covered with gelatinous deposits. On the serosal surface of the fallopian tubes, similar jelly-like masses were identified. The appendix measured 5 cm in length and approximately 1 cm in diameter, with focal deposits of colloid-like material on its serosal surface. The gross appearance was consistent with mucinous involvement with possible peritoneal dissemination.

**Figure 10:**
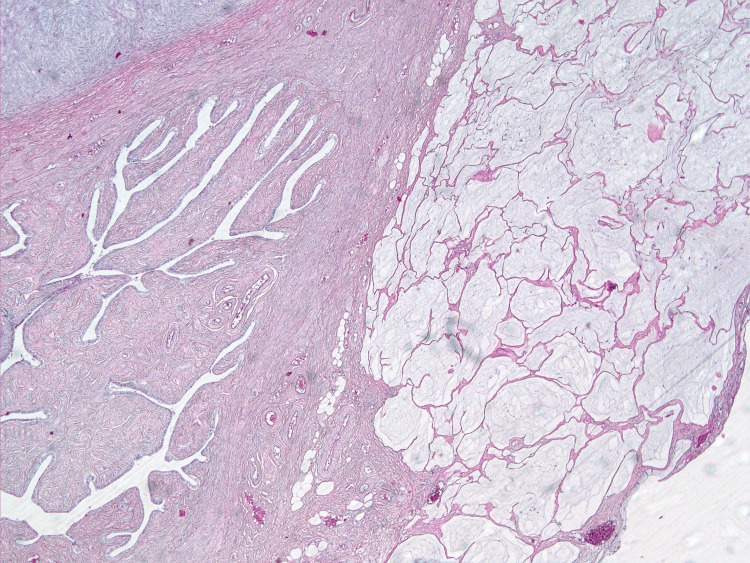
Masses of cell-free mucin on the serous membrane of the fallopian tube. Hematoxylin and eosin staining, ×10 magnification.

**Figure 11:**
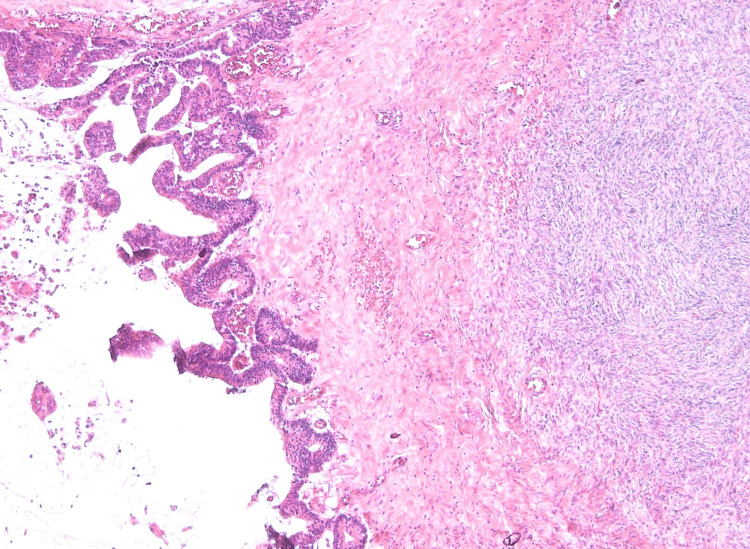
The focus of tumor growth on the ovarian capsule with intussusception deep into the parenchyma. Hematoxylin and eosin staining, ×10 magnification.

**Figure 12:**
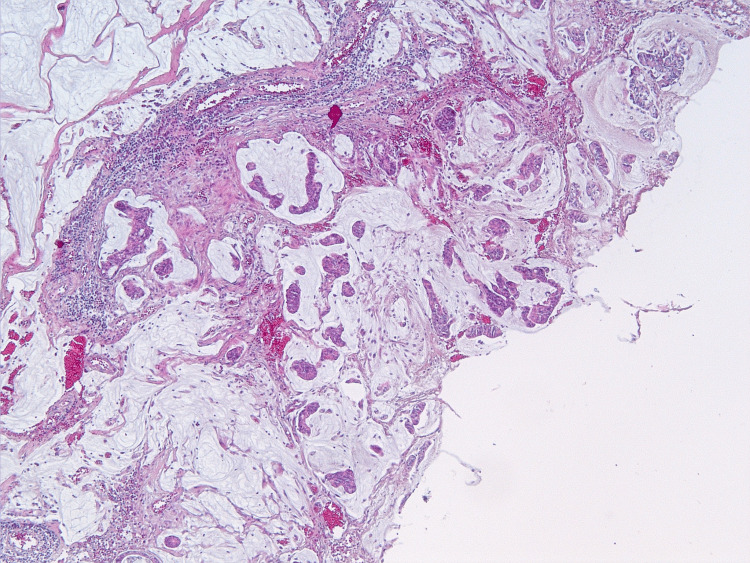
Tumor growth on the serous membrane of the appendix. Hematoxylin and eosin staining, ×10 magnification.

**Figure 13:**
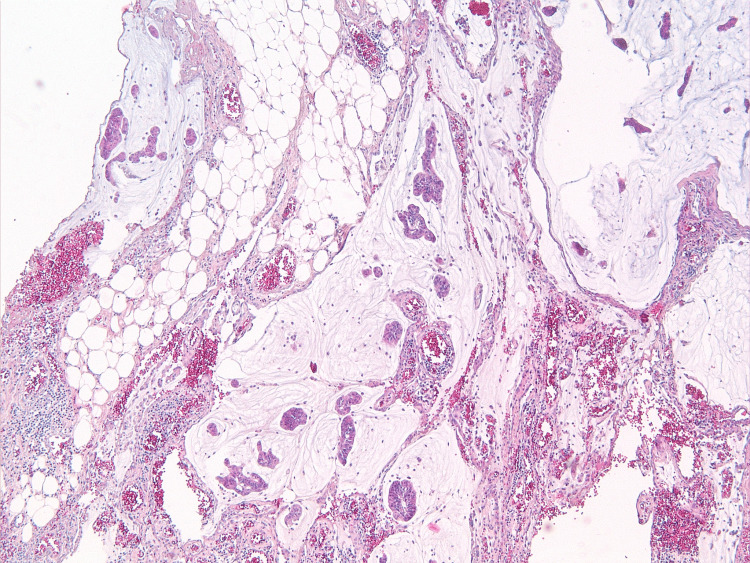
The focus of tumor growth in the large omentum. Hematoxylin and eosin staining, x20 magnification.

**Figure 14:**
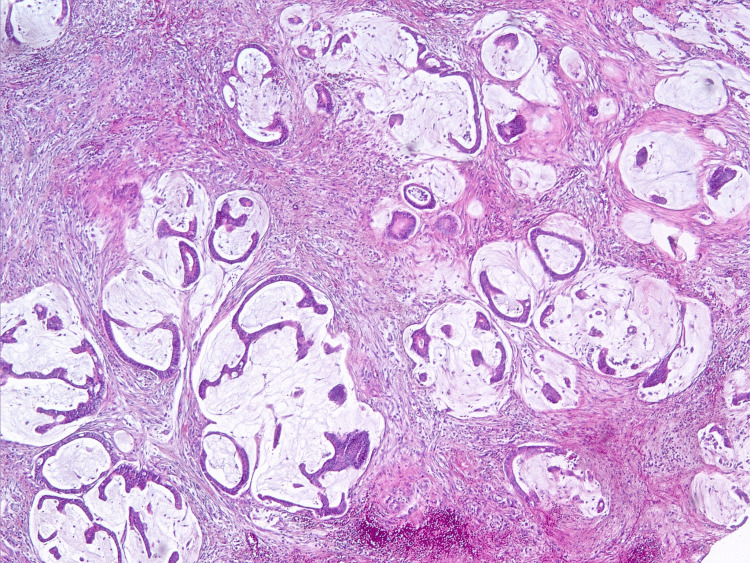
The tumor is represented by lakes of extracellular mucus with free-lying layers of tumor cells with signs of moderate cytological atypia. Hematoxylin and eosin staining, ×10 magnification.

**Figure 15:**
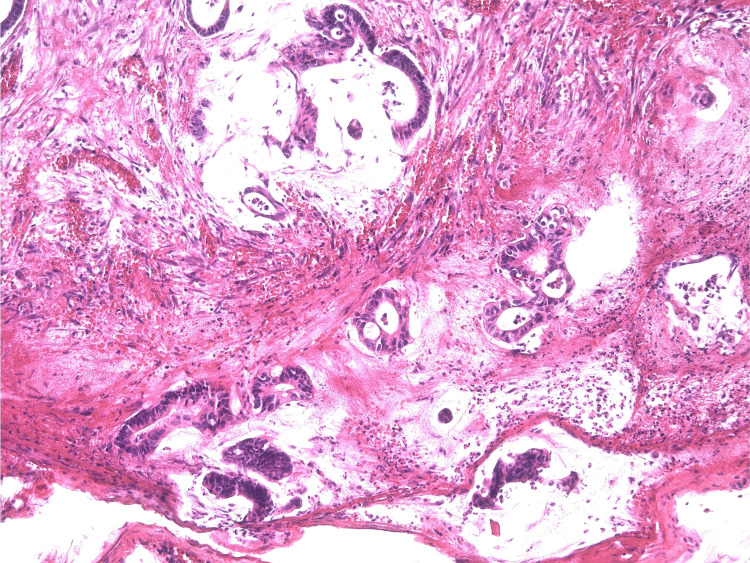
The focus of infiltrating growth (peritoneum of the Morrison pocket), represented by angular glands with a perifocal desmoplastic stroma reaction. Hematoxylin and eosin staining, ×20 magnification.

On microscopic examination, the parietal and visceral peritoneum, as well as the tissues of the greater and lesser omentum, showed massive accumulations of extracellular mucin, within which sheets and clusters of tumor cells were freely floating. The cells were columnar and cuboidal in shape, with moderately eosinophilic cytoplasm and ovoid nuclei containing dispersed chromatin and inconspicuous nucleoli. Moderate nuclear polymorphism was noted. In some areas (particularly on the peritoneum of Morison's pouch and in the greater omentum), glandular structures exhibiting infiltrative growth were found; these glands had angular shapes and were surrounded by a prominent desmoplastic stromal reaction. Focal tumor involvement was also documented on the serosa of the appendix and the small intestinal wall fragments, while the mucosa remained intact. Ovarian involvement was limited to mucinous deposits and sheets of tumor cells on their capsules, with involvement of the fallopian tube serosa. The internal structure (parenchyma) of both ovaries showed scleroatrophic changes and corpora albicantia; no evidence of a primary ovarian tumor was found. Metastases consisting of pools of acellular mucin were identified in the hepatoduodenal ligament lymph nodes (Figures 10-15).

On immunohistochemical (IHC) analysis, the tumor cells showed positive expression for CK20 and CDX2 markers. Expression of CK7 and WT1 was negative. It strongly supports a gastrointestinal origin, as primary ovarian mucinous tumors typically exhibit a different profile, such as CK7 positivity and CDX2 negativity. The p53 status corresponded to a wild-type expression pattern, characteristic of low-grade neoplasms.

Considering the morphological findings, the IHC profiling results, the intraoperative pattern of spread, and the imaging data, the final pathological diagnosis was formulated as: Mucinous carcinoma with spread consistent with pseudomyxoma peritonei, Grade 2 according to the WHO Classification of Tumours of the Female Reproductive System (5th edition, 2020) [12]. Histologically, features supporting Grade 2 classification included infiltrative-type tumor invasion (presented as angulated glands in a desmoplastic stroma in some metastatic sites), areas of hypercellular mucinous deposits, and the finding that more than 10% of the tumor exhibited high-grade cytologic features.

Follow-up

At the time of writing this article (May 2025), 12 months after surgical treatment, the patient is under active surveillance. There are no clinical, laboratory, or radiological signs of disease recurrence or progression.

## Discussion

PMP is an extremely rare clinicopathological condition characterized by the accumulation of mucinous material within the abdominal and pelvic cavities. According to various epidemiological data, the incidence of PMP is approximately 1-2 cases per million people per year [13], which underscores both the relevance and complexity of diagnosing and managing this disease. Despite many years of research efforts, the etiology and pathogenesis of PMP remain subjects of active investigation and discussion. It is believed that mucin-producing low-grade appendiceal mucinous neoplasms, which may remain latent for a long period, can eventually rupture due to increasing intratumoral pressure and capsule overdistension. This, in turn, leads to the dissemination of tumor cells and mucin throughout the peritoneal cavity [14].

A distinctive feature of PMP is the predominance of extracellular mucin in the tumor substrate. The ratio of the mucinous component to tumor cells can reach 10:1 [15], explaining the specific clinicopathological presentation of the disease. Despite extensive peritoneal spread, distant organ metastasis is extremely rare in PMP, a point emphasized by several authors [2,11,16].

The issue of the primary origin of PMP remains a subject of scientific debate. For a long time, the ovaries were considered a possible site of origin for the disease, particularly in women. However, more recent studies based on morphological and immunohistochemical analysis provide evidence favoring the appendix as the most likely primary source of the mucinous neoplasia. For instance, in the work by Ronnett et al. [11], conducted on a sample of 30 women diagnosed with PMP, it was shown that in the vast majority of cases, the source of the mucinous tumor was located in the appendix or the intestine. Although PMP is traditionally considered a neoplastic process of appendiceal origin [11,14,17], the literature contains descriptions of rare cases of PMP arising from other anatomical sites, including the ovaries, colon, and even the pancreas [2,5,9,11,14,15,17,18]. These findings highlight the heterogeneity of the disease and the necessity of a comprehensive diagnostic approach involving morphological, immunohistochemical, and radiological methods.

The term PMP encompasses a spectrum of peritoneal mucinous dissemination, ranging from acellular mucin deposits with indolent behavior to highly aggressive forms with high-grade invasive tumor cells. Consequently, using the single term “PMP” may be inadequate for diagnostic and prognostic precision. To differentiate the morphological variants of PMP, a classification based on histological features, distinguishing two main categories: disseminated peritoneal adenomucinosis (DPAM) and PMCA [11]. Disseminated peritoneal adenomucinosis (DPAM) is characterized by abundant extracellular mucin with scant strips of mucin-producing epithelium exhibiting low mitotic activity and minimal cytological atypia. Whereas PMCA involves peritoneal lesions with a more prominent epithelial component, higher cellular density, features of invasive growth, and marked atypia typical of adenocarcinoma.

In 2010, the WHO, in collaboration with the American Joint Committee on Cancer (AJCC), proposed an updated classification for PMP based on morphological, cytological, and molecular features, dividing the disease into low-grade and high-grade forms [19]. According to these criteria, establishing the diagnosis of PMP requires both clinical and morphological confirmation: the presence within the peritoneal cavity of diffuse mucin collections and mucinous implants, as well as the histological identification of mucin-producing neoplastic epithelial cells. In the absence of an epithelial component in the examined material, using the term “mucinous ascites” rather than PMP is considered more accurate [14,20]. From a molecular pathogenesis perspective, goblet cells of the appendix play an important role in the development of PMP. These cells possess the ability to produce mucin and form tight intercellular junctions with the surrounding stroma. Studies have shown that such cells selectively express the *MUC2 *gene, which encodes one of the mucins and serves as a highly specific marker for PMP of appendiceal origin. These findings, first presented by O'Connell et al. [1], strongly support the theory of the appendix's primary role as the source of the primary tumor in PMP, as opposed to the hypothesis of ovarian origin.

PMP is more frequently diagnosed in women. The most characteristic clinical manifestation of the disease is an increase in abdominal girth and a feeling of discomfort, caused by the accumulation of mucinous ascites within the peritoneal cavity. In progressive cases, the phenomenon known as “jelly belly” develops, accompanied by pronounced abdominal distension and palpable soft tissue masses [13,15,17,20]. However, in cases with less extensive involvement, the clinical picture may be limited to non-specific abdominal pain without obvious abdominal distension, which can mimic acute appendicitis and lead to diagnostic errors [15,20]. Additionally, the elevated intra-abdominal pressure characteristic of PMP can provoke the development of ventral hernias or pelvic organ prolapse. Consequently, the disease is sometimes detected incidentally during surgical interventions performed for these indications. In some female patients, PMP may present as a palpable pelvic mass, requiring differential diagnosis from tubo-ovarian abscess or malignant tumors of the pelvic organs [2,20,21].

CT allows for the identification of characteristic signs of the disease, including diffuse low-density mucinous ascites in the abdominal and pelvic cavities, multiple septations, and calcifications. Liver scalloping is a highly characteristic radiologic sign that helps distinguish PMP from simple free ascites [15, 20, 21].

With a significant volume of tumor burden in the abdominal cavity, visualization of the appendix on CT is often impossible [21]. Even with a smaller volume of disease, the appendix was clearly visualized only in a limited number of patients. Moreover, the CT scan findings correlated well with intraoperative findings.

The anatomical distribution of tumor cells and mucin in PMP is determined by factors such as the flow and absorption of peritoneal fluid, as well as the effects of gravity. As a result, preferential accumulation of tumor material is observed in specific anatomical regions of the abdominal cavity: the omentum (leading to the formation of a characteristic “omental cake”), the region of the right diaphragmatic dome, the right retrohepatic space, the left paracolic gutter, and the pelvis. Conversely, other areas of the peritoneum are involved much less frequently [4,9,11,15,17,21].

The diagnostic value of serum tumor markers - CA-125, CA 19-9, and CEA - remains limited in PMP. However, they have some value in monitoring patients during the postoperative period and can serve as indicators of disease recurrence [13,15,22]. Before the introduction of HIPEC, therapeutic approaches for PMP were limited to repeated debulking surgical procedures. The introduction of HIPEC represented a significant breakthrough in the treatment of peritoneal malignancies, including PMP, and has substantially improved clinical outcomes [2,9,15,18,23]. Sugarbaker and Chang [4], in a study involving 385 patients with appendiceal tumors spread to the peritoneum, demonstrated that the combination of CRS with HIPEC achieved 5-year survival rates of up to 86% in patients with adenomucinosis (low-grade PMP). The optimal approach is considered to be CRS involving parietal and visceral peritonectomy, combined with intraoperative HIPEC. However, such interventions are characterized by high technical complexity: the average operative time is approximately 10.5 hours, and they are associated with increased risks of postoperative complications and mortality [13]. The exclusive use of systemic chemotherapy is not recommended for PMP due to its limited efficacy, attributed to poor drug perfusion through the mucinous matrix and insufficient penetration into the tumor tissue. According to data from Kojimahara et al. [23], systemic chemotherapy does not significantly impact overall survival in PMP patients, whereas intraperitoneal administration of chemotherapy is associated with better oncological outcomes. Thus, systemic chemotherapy may be considered an option only in cases where the patient is not a candidate for CRS followed by HIPEC-for example, due to severe comorbidities, poor performance status, or limited access to specialized medical care [2,9,15,20,22,23]. In these situations, repeat palliative CRS may also be justified with the aim of reducing tumor bulk and improving the patient's quality of life.

According to data presented by Wheeler et al. [24], in patients with disseminated peritoneal adenomucinosis (DPAM), performing CRS alone without subsequent HIPEC is associated with a less favorable prognosis, although it does not significantly impact overall survival. The authors note that adding HIPEC may increase treatment toxicity and its overall burden for patients with DPAM. Nevertheless, for PMCA, CRS combined with HIPEC remains the most appropriate approach.

The prognosis for patients with PMP depends on several key factors, including the volume of tumor involvement, the CC, and the differentiation grade of the tumor cells. Despite comprehensive treatment, patients with elevated preoperative tumor marker levels generally have a higher risk of disease recurrence [13,14,22]. An increase in the concentration of markers such as CEA, CA 19-9, and CA 125 during follow-up significantly correlates with the development of recurrence, whereas consistently low marker levels are associated with a favorable prognosis [13,14,15,20,22].

Five-year and ten10-year survival rates for patients undergoing standard CRS are 53-75% and 32-60%, respectively. However, aggressive cytoreduction combined with HIPEC can increase 10-year survival to up to 90% [15].

Interest in alternative treatment methods for PMP also persists. Fiorelli et al. demonstrated in vitro cytotoxic activity of dilute povidone-iodine (PVP-I, Betadine®) against malignant pleural mesothelioma cell lines, with maximal effect at 0.1% - far below the 10% concentration used clinically as a skin antiseptic [25]. Although these laboratory findings raise a theoretical possibility of intraperitoneal lavage with low-concentration PVP-I in patients who cannot undergo radical CRS and HIPEC, such an approach remains entirely experimental. Potential complications-including peritoneal inflammation, adhesions, iodine absorption, thyroid dysfunction, and nephrotoxicity-would need to be carefully investigated in pre-clinical safety studies before any clinical application.

## Conclusions

PMP remains a rare and complex disease that often presents significant diagnostic challenges. Although numerous attempts have been made to establish an optimal treatment protocol, many approaches have failed to deliver satisfactory long-term outcomes.

This case report confirms that complete CRS with hyperthermic intraperitoneal chemotherapy (HIPEC) provides the best potential for favorable outcomes. However, there are significant challenges regarding optimal patient selection for this therapy and improving treatment accessibility. Therefore, further investigation is needed to develop effective therapeutic options for a broader range of PMP patients, including those who may not be candidates for extensive surgery and HIPEC.
